# CXCL12 and [N33A]CXCL12 in 5637 and HeLa Cells: Regulating HER1 Phosphorylation *via* Calmodulin/Calcineurin

**DOI:** 10.1371/journal.pone.0034432

**Published:** 2012-04-18

**Authors:** Antonella Rigo, Michele Gottardi, Ernesto Damiani, Massimiliano Bonifacio, Isacco Ferrarini, Pierluigi Mauri, Fabrizio Vinante

**Affiliations:** 1 Department of Medicine, Section of Hematology, University of Verona, Verona, Italy; 2 Department of Experimental Biomedical Sciences, University of Padua, Padua, Italy; 3 Proteomics and Metabolomics Unit, Institute for Biomedical Technologies, CNR, Milan, Italy; Cincinnati Children's Hospital Medical Center, United States of America

## Abstract

In the human neoplastic cell lines 5637 and HeLa, recombinant CXCL12 elicited, as expected, downstream signals *via* both G-protein-dependent and β-arrestin-dependent pathways responsible for inducing a rapid and a late wave, respectively, of ERK1/2 phosphorylation. In contrast, the structural variant [N33A]CXCL12 triggered no β-arrestin-dependent phosphorylation of ERK1/2, and signaled *via* G protein-dependent pathways alone. Both CXCL12 and [N33A]CXCL12, however, generated signals that transinhibited HER1 phosphorylation *via* intracellular pathways. 1) Prestimulation of CXCR4/HER1-positive 5637 or HeLa cells with CXCL12 modified the HB-EGF-dependent activation of HER1 by delaying the peak phosphorylation of tyrosine 1068 or 1173. 2) Prestimulation with the synthetic variant [N33A]CXCL12, while preserving CXCR4-related chemotaxis and CXCR4 internalization, abolished HER1 phosphorylation. 3) In cells knockdown of β-arrestin 2, CXCL12 induced a full inhibition of HER1 like [N33A]CXCL12 in non-silenced cells. 4) HER1 phosphorylation was restored as usual by inhibiting PCK, calmodulin or calcineurin, whereas the inhibition of CaMKII had no discernable effect. We conclude that both recombinant CXCL12 and its structural variant [N33A]CXCL12 may transinhibit HER1 *via* G-proteins/calmodulin/calcineurin, but [N33A]CXCL12 does not activate β-arrestin-dependent ERK1/2 phosphorylation and retains a stronger inhibitory effect. Therefore, we demonstrated that CXCL12 may influence the magnitude and the persistence of signaling downstream of HER1 in turn involved in the proliferative potential of numerous epithelial cancer. In addition, we recognized that [N33A]CXCL12 activates preferentially G-protein-dependent pathways and is an inhibitor of HER1.

## Introduction

CXCL12 regulates important hematopoietic functions, induces cell adhesion and chemotaxis, and coordinates the circulation of hematopoietic stem cells, lymphocytes and monocytes [1], [2], [3]. Stromal and endothelial cells constitutively express CXCL12 in bone marrow, lymph nodes, liver, lung and skin [4]. As far as cancer growth is concerned, CXCL12 may induce mitotic signals, favor metastatic evolution and contribute to the development of a microenvironment dominated by M2-polarized, anti-inflammatory, tumor-associated macrophages that support tumor cell survival [5], [6], [7], [8]. Notably, cancer cells usually express functional receptors for CXCL12 and some cancers constitutively express CXCL12 [2], [7], [8].

**Figure 1:**
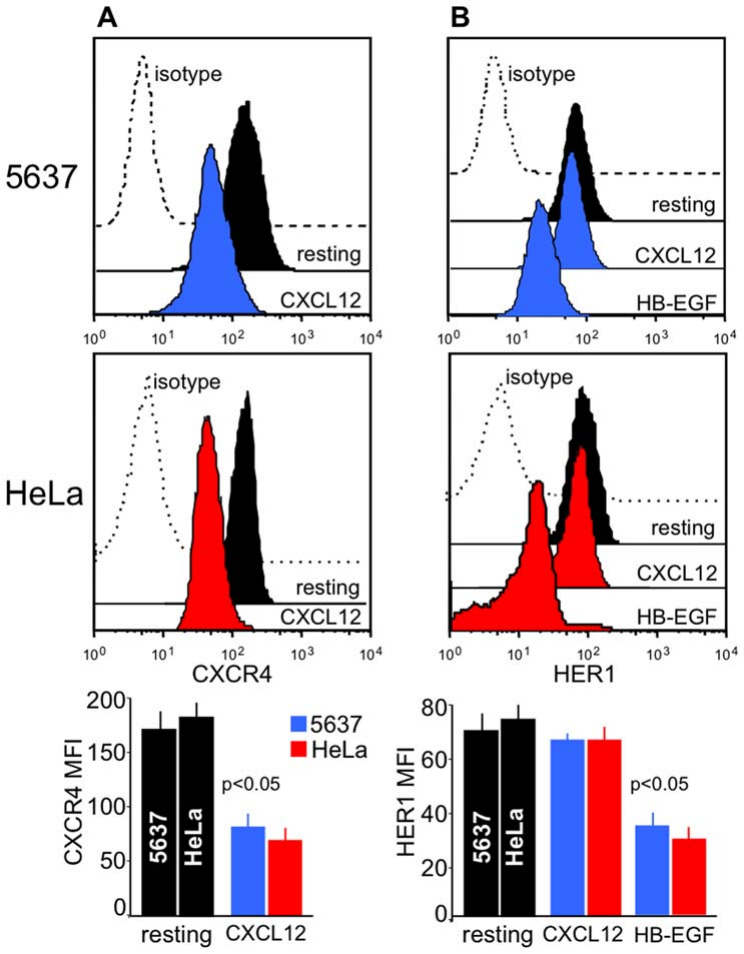
Receptors involved in the crosstalk between CXCL12 and HER1. 5637 or HeLa cells were cultured alone, or in the presence of 200 ng/mL CXCL12 or 25 ng/mL HB-EGF. After 20 minutes, the cells were collected and evaluated by flow cytometry for the expression of CXCR4 and HER1. (A) Stimulation with CXCL12 led to downregulation of CXCR4 due to its internalization. (B) Stimulation with HB-EGF induced HER1 internalization. In contrast, no changes in the surface expression of HER1 were detected after stimulation with CXCL12. Thus, CXCL12 binding to CXCR4 did not transactivate HER1 in these cells *via* shedding of HER1 ligands. Representative flow cytometry patterns and the means ±SD of 10 experiments are depicted.

CXCL12 elicits cellular responses by binding to CXCR4 and CXCR7, which are seven transmembrane receptors (7TMR) that activate G-proteins [9]. Though the role of CXCR7 is not fully understood up to date [10], [11], it is known that downstream of CXCR4 native CXCL12 activates both G-protein-dependent and β-arrestin-dependent signaling pathways, which may support distinct intracellular messages [12], [13], [14]. It seems that structural variants of either the ligand or the receptor may result in the dissociated activation of either G-protein- or β-arrestin-dependent pathways, followed by different cellular responses [13], [15], [18].

By binding to its receptors, CXCL12 induces another interesting phenomenon, i.e. the transactivation of the human EGF receptor 1 (HER1) and other HER family members. Such a crosstalk is a general function of 7TMR signaling [19], including CXCR4 [8]. Usually the transactivation occurs in a paracrine manner. For instance, the ligation of CXCR4 activates membrane metalloproteinases, which release HER1 ligands such as EGF or HB-EGF from cell membrane resulting in their binding to HER1 [8], [19], [20], [21]. This induces kinase-dependent autophosphorylation of cytoplasmic tail residues on HER1 leading to a signaling cascade that is involved in the proliferation, morphogenesis and differentiation of both normal and neoplastic cells [22]. HER1 is often expressed by epithelial cancer and plays a role in tumor progression by inducing proliferation, resistance to growth-inhibitory cytokines and the expression of selective immunosuppressive cytokines, proangiogenic cytokines, and chemokines [23].

In HERs/CXCR4 double-positive cells, a transactivation of HERs along intracytoplasmic pathways is possible and was described [24]. Indeed, intracytoplasmic transactivation was shown to be important in breast cancer [25] and myeloma progression [26]. Here we provide the unexpected evidence that CXCL12 may transhinibit HER1. Using the bladder cancer cell line 5637 and the cervical cancer cell line HeLa, which is an established *in vitro* model for studying both HER1 and CXCR4 signaling [4], [27], we found that CXCL12 elicits a calmodulin/calcineurin pathway, thereby delaying the autophosphorylation peak of HER1. Remarkably, we observed that the fully active, synthetic mutant [N33A]CXCL12 [28] was biased for G- protein-dependent pathways failing to activate β-arrestin-dependent ERK1/2 phosphorylation downstream of CXCR4 and could completely, though transiently, block HER1 phosphorylation.

## Materials and Methods

### Cells

The 5637 (bladder carcinoma) and HeLa (cervical carcinoma) human cell lines, purchased from American Type Culture Collection, were grown in DMEM+10% FCS (Invitrogen, Carlsbad, CA) until confluence and were subsequently detached by accutase (Innovative Cell Technologies, San Diego, CA).

**Figure 2:**
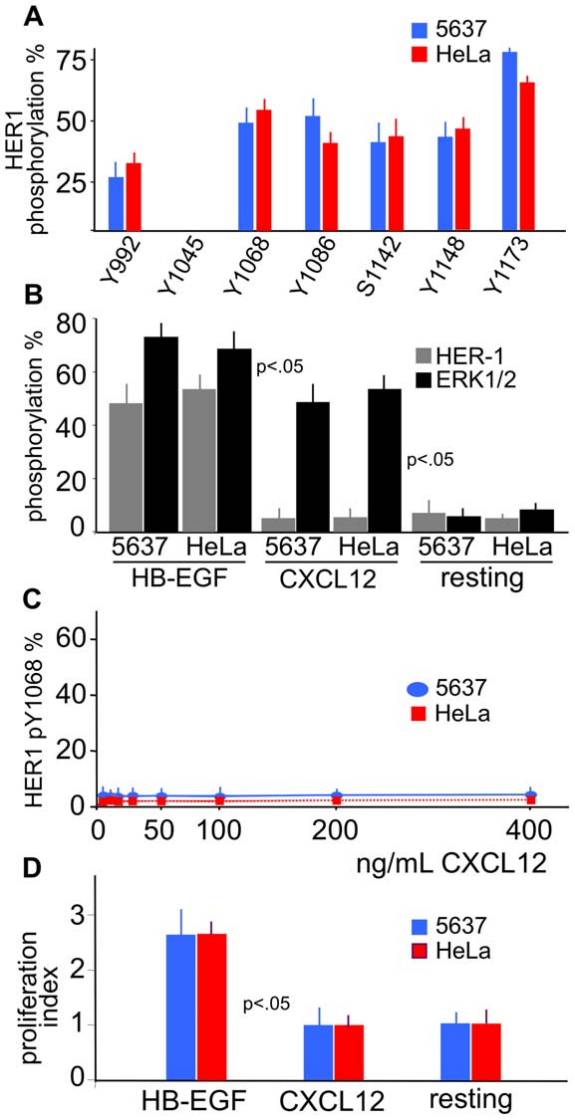
Phosphorylation of HER1 and ERK1/2, and cell proliferation following treatment with HB-EGF and CXCL12. (A) 5637 and HeLa cells were stimulated with 25 ng/mL HB-EGF for 20 minutes, and cell lysates were analyzed by mass spectrometry following trypsin digestion. Y1068 and Y1173, sites of autophosphorylation coupled to the activation of Ras, MEK and ERK1/2, were evaluated in the following experiments with specific phosphotyrosine mAbs. (B) HB-EGF induced phosphorylation of both HER1 Y1068 and ERK1/2 TY185/187 in 5637 or HeLa cells. In contrast, stimulation with 200 ng/mL CXCL12 for 20 minutes, which induced phosphoERK1/2, led to no phosphorylation of HER1. (C) In dose-response experiments, exposures of CXCL12 ranging from 6 to 400 ng/mL for 20 minutes did not induce HER1 phosphorylation in either cells. (D) No proliferation was induced with 200 ng/mL CXCL12. The means ±SD of 10 experiments are depicted.

### Flow cytometry

Cells under basal conditions or following stimulation for 20 minutes with 200 ng/mL CXCL12 (Peprotech, London, UK) or 25 ng/mL HB-EGF (R&D Systems, Minneapolis, MN) were detached and incubated with anti-human PE-tagged CXCR4 or HER1 mAbs, as well as appropriate isotype controls (BD Pharmingen, San Jose, CA). Cells were analyzed on a FACSCalibur (Becton-Dickinson, Mountain View, CA) flow cytometer running FlowJo 8.8.2 software (Tree Star, Ashland, OR).

**Figure 3:**
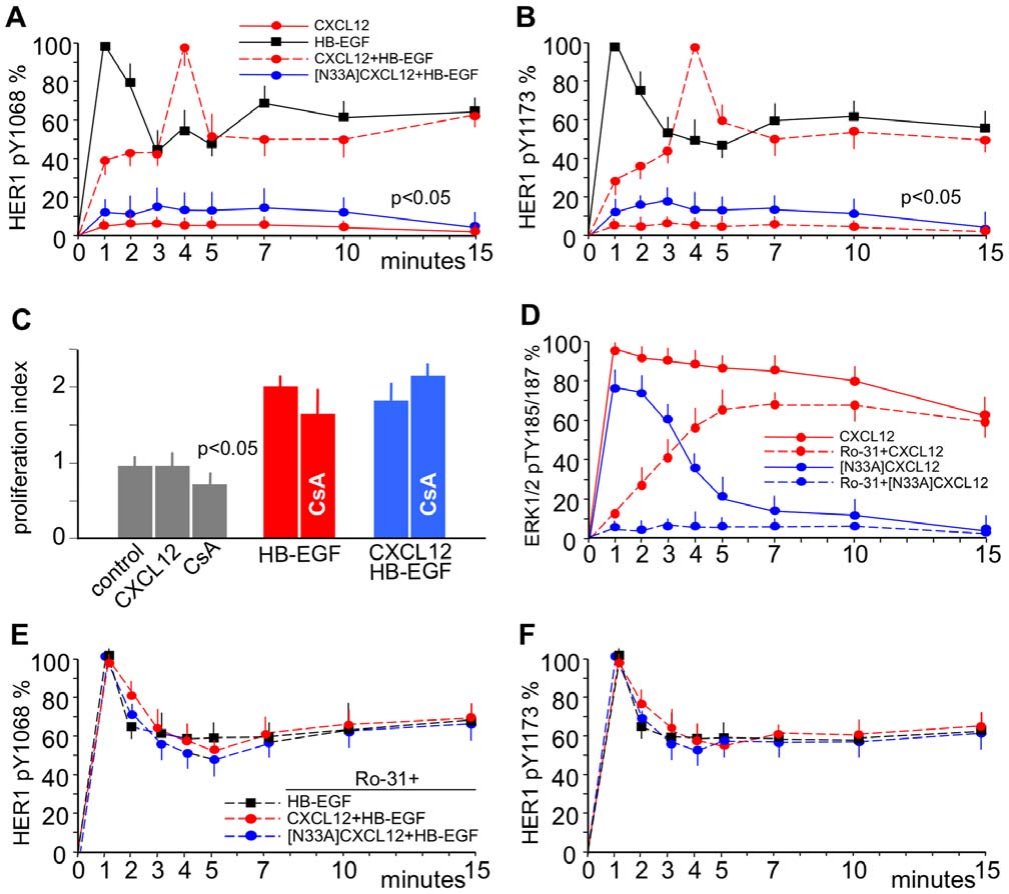
CXCL12 regulates HER1 Y1068 and Y1173 phosphorylation via G-proteins. 5637 or HeLa cells were stimulated with 25 ng/mL HB-EGF or with 200 ng/mL CXCL12 or N33A]CXCL12 alone or followed after 1 minute by 25 ng/mL HB-EGF. HER1 Y1068 or ERK1/2 TY185/187 phosphorylation was evaluated at the indicated time-points by using specific mAbs and was expressed as percentages of phosphorylation at the specified times after normalization as phosphorylated molecule/total molecule ratios. (A) HER1 phosphorylation at Y1068 in HeLa cells induced by HB-EGF alone (black) was abolished by prestimulation with [N33A]CXCL12 (blue) and modified by prestimulation with CXCL12 (red): maximum phosphorylation was reached at 4 minutes, and the plateau after the initial spike was around 50% of the maximum at 10 minutes. No phosphorylation was induced by CXCL12 alone. (B) Phosphorylation at Y1173 in 5637 cells displayed the same kind of pattern. (C) Prestimulation with CXCL12 did not modify the mitogenic effect of HB-EGF. (D) Stimulation with CXCL12 induced ERK1/2 phosphorylation (red) resulting from two spikes: a G-protein-dependent (blue) and a β-arrestin-dependent (red) phosphorylation spike. By using the PKC inhibitor Ro-31 the G-protein-dependent spike was abolished, whereas the β-arrestin-dependent spike persisted. [N33A]CXCL12 induced only the G-protein-dependent spike (blue), which was abolished by Ro-31. (E) Ro-31 abolished the effects of prestimulation with CXCL12 or [N33A]CXCL12 at Y1068 in 5637 cells. (F) The same pattern at Y1173 in HeLa cells. The means ±SD of 10 experiments are depicted.

### HER1 stimulation

Semiconfluent, 24-hour starved cells were incubated in the presence or absence of 25 ng/mL recombinant human HB-EGF or 200 ng/mL of either recombinant human CXCL12 or chemically synthesized [N33A]CXCL12 (BD PharMingen, San Diego, CA). In selected experiments, HeLa cells were prestimulated for 1 minute with either 200 ng/mL recombinant human CXCL12 (BD PharMingen) or chemically synthesized [N33A]CXCL12 and subsequently stimulated with 25 ng/mL HB-EGF. Cell pellets were obtained to perform protein extractions at different treatment times ranging from 1 to 15 minutes.

**Figure 4:**
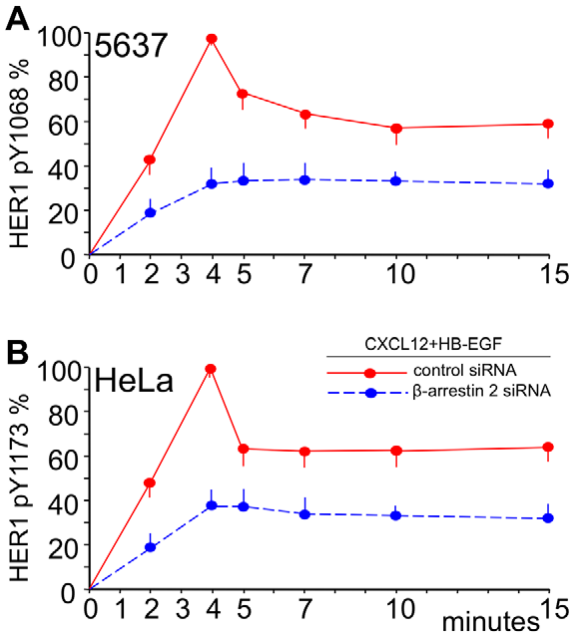
Knockdown of β-arrestin 2 protein levels allows for transinhibition of HER1. 5637 or HeLa cells were transfected with control siRNA or β-arrestin 2 siRNA, prestimulated for 1 minute with 200 ng/mL CXCL12 and subsequently stimulated with 25 ng/mL HB-EGF for 2, 4, 5, 7, 10 or 15 minutes. In knockdown cells either Y1068 or Y1173 phosphorylation was inhibited as opposed to non-silenced cells at each time observed, as determined by ELISA (p<0.05). This shows that CXCL12 signaling transinhibits HER1 phosphorylation via G-protein-pathways in the absence of β-arrestin 2 activation and further supports that [N33A]CXCL12, which strongly transhinibits HER1, is a G-protein-biased ligand. The means ±SD out of 4 experiments are shown.

### Protein extraction

Cell pellets were lysed for 30 minutes in 1 mL of ice-cold cell extraction buffer (Biosource, Camarillo, CA) that was supplemented with a protease inhibitor cocktail (Sigma, St. Louis, MO) and 1 mM PMSF (Sigma). After centrifugation at 13,000 rpm for 10 minutes at 4°C, aliquots of supernatants were stored at −80°C until used.

### ELISA

Total HER1 and HER1 pY1068 or pY1173, and total ERK1/2 and ERK1/2 pTY185/187 were evaluated in cell protein extracts by using commercially available ELISA kits (Biosource). The results were expressed as percentages of phosphorylation at the specified times after normalization as phosphorylated molecule/total molecule ratios.

**Figure 5:**
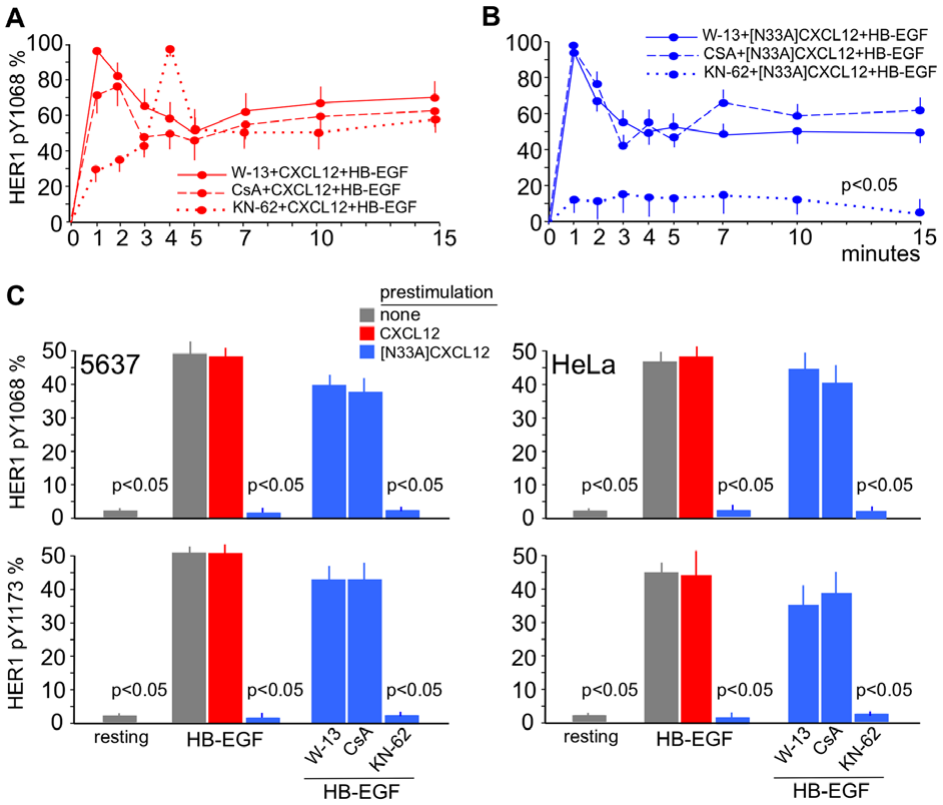
Regulation of HER1 Y1068 or Y1173 phosphorylation downstream of G-proteins requires calmodulin/calcineurin activity. (A) and (B) Time-course. HeLa cells were stimulated as indicated in Figure 3. Treatment with W-13 (a calmodulin inhibitor) or CsA (a calcineurin inhibitor) restored HB-EGF-dependent HER1 phosphorylation at Y1068, whereas KN-62 (a CaMKII inhibitor) had no effect. (C) Following prestimulation with [N33A]CXCL12 the HB-EGF-dependent HER1 phosphorylation at Y1068 and Y1173 was abolished at the plateau (15 minutes) and restored by W-13 or CsA; KN-62 had no effect. The means ±SD of 10 experiments are depicted.

### Mass spectrometry

Around 1×10^7^ cells, either unstimulated or stimulated with 25 ng/mL HB-EGF for 15 minutes, were harvested and underwent HER1 immunoprecipitation, reduction, alkylation and tryptic digestion. Ten microliter aliquots of each resulting peptide mixture were analyzed by LC-MS/MS (liquid chromatography coupled to an ion trap mass spectrometer) system [29]. Data handling for the identification of phosphorylated residues was performed according to the method of Guo et al. [30]. HB-EGF-induced HER1 phosphorylation at Y992, Y1045, Y1068, Y1086, Y1148, Y1173 and S1142 was evaluated.

### Proliferation of cells

Approximately 2,500 cells/well were seeded in 96-well plates and allowed to adhere for 24 hours, prior to incubation with 25 ng/mL HB-EGF and/or 200 ng/mL recombinant human CXCL12 and/or 1 µg/mL cyclosporin A (CsA, Novartis, Origgio, Italy). After 72 hours, the proliferation of stimulated *vs*. unstimulated cells was measured by MTT [3-(4, 5-dimethyl-thiazol-2-yl)-2,5-diphenyl tetrazolium bromide] incorporation, as previously described [31].

### siRNA transfection

To silence the expression of β-arrestin 2, 5637 or HeLa cells were seeded on 12-well plates (1.2×10^5^) and transfected 72 hours using HiPerFect Transfection Reagent and siRNA (2 nM, β-arrestin 2 siRNA no. SI02776928 and AllStars negative control siRNA – Alexa Fluor 488; Qiagen, Hilden, D) as previously described [8]. Transfection efficiency (>95%) was determined by flow cytometry on trypsinized cells. At the end of colture, cells were starved for 4 hours, prestimulated for 1 minute with 200 ng/mL recombinant human CXCL12 and then with 25 ng/mL HB-EGF for 2, 4, 5, 7, 10 or 15 minutes. Cell pellets were obtained to perform protein extractions and ELISA for total HER1 and HER1 pY1068 or pY1173 as reported above.

### Transduction pathway inhibition

Semiconfluent, 24-hour starved cells were exposed for 30 minutes to 5 µM 2-{1-[3-(Amidinothio)propyl]-1H-indol-3-yl}-3-(1-methylindol-3-yl)maleimide methanesulfona-te salt (Ro-31-8220; Sigma-Aldrich) or 44 µM N-(4-Aminobutyl)-5-chloro-2-naphthalenesulfonamide hydrochloride (W-13; Sigma-Aldrich),) or 30 µM 1-[N, O–bis-(5-Isoquinolinesulfonyl)–N–methyl-L-tyrosyl]-4-phenylpiperazine (KN-62; Calbiochem, La Jolla, CA) or 1 µg/mL Cyclosporin A (CsA; Sigma-Aldrich),) – specific inhibitors of PKC, calmodulin, CaMKII and calcineurin, respectively. HeLa cells were then treated with 200 ng/mL human recombinant CXCL12 (BD PharMingen) or chemically synthesized [N33A]CXCL12 (BD PharMingen) for up to 15 minutes or for 1 minute before stimulation with 25 ng/mL HB-EGF for up to 15 minutes.

### Statistics

Student's t-test, Mann-Whitney U test and Kruskall-Wallis ANOVA by ranks were used. Differences were considered significant for p values <0.05.

## Results

### CXCL12 does not transactivate HER1 in 5637 or HeLa cells

Like other epithelial cells, 5637 and HeLa cells co-express HER1 and CXCR4 under basal conditions (Figure 1A, B). For stimulation of 7TMRs, such as CXCR4, leads to metalloprotease-induced shedding of EGF-family ligands, we wondered if CXCL12, the ligand of CXCR4, could transactivate HER1 in 5637 or HeLa cells. As shown in Figure 1A, stimulation of 5637 or HeLa cells with 200 ng/mL CXCL12 led to CXCR4 downregulation, which implies that CXCL12 bound to its receptors and triggered internalization. In contrast, HER1 was not downregulated following CXCL12 stimulation (Figure 1B). When cells, however, were stimulated with 25 ng/mL HB-EGF, HER1 was induced to internalize following ligand binding (Figure 1B). Because HER1 transactivation *via* binding of a shedded ligand would ultimately be indicated by HER1 internalization, we concluded that CXCL12 did not induce HER1 transactivation in 5637 or HeLa cells through this mechanism. Another approach implies evaluating the phosphorylation of HER1 and downstream of HER1 associated with different stimuli.

### HER1 phosphorylation in 5637 and HeLa cells

To demonstrate the full sensitivity of 5637 and HeLa cells to HER1 stimulation, we assessed the full pattern of HB-EGF-induced HER1 phosphorylation at tyrosine (Y992, Y1045, Y1068, Y1086, Y1148, Y1173) and serine (S1142) residues by mass spectrometry analysis of trypsin-digested peptides. The results are depicted in Figure 2A. After treatment with 25 ng/mL HB-EGF for 20 minutes, there was no phosphorylation of Y1045, while the remaining tyrosine sites showed different degrees of phosphorylation. Based on these results, we routinely assessed the phosphorylation status of the HER1 tyrosine 1068 and 1173, two major site of autophosphorylation related to the activation of Ras, MEK and ERK1/2. The phosphorylation of tyrosine 1068 (pY1068) and 1173 (pY1173) is followed by downstream ERK1/2 phosphorylation at threonine 185 and tyrosine 187 (pTY185/187) [22].

### HER1 and ERK1/2 phosphorylation in 5637 and HeLa cells

Then, we analyzed the phosphorylation status of HER1 Y1068 and of ERK1/2 T185 and Y187 following stimulation of 5637 or HeLa cells with 25 ng/mL HB-EGF or 200 ng/mL CXCL12 for 20 minutes. Figure 2B shows that HB-EGF induced the phosphorylation of HER1 at Y1068 as well as ERK1/2 at T185 and Y187. By contrast, treatment with CXCL12 phosphorylated ERK1/2 but did not induce any HER1 phosphorylation. In addition, treatment with different concentrations of CXCL12 (up to 400 ng/mL) for different amounts of time (1 up to 20 minutes) did not phosphorylate HER1 (Figure 2C). Finally, CXCL12 did not lead to cell proliferation, whereas HB-EGF was a mitogenic agent (Figure 2D). Thus, CXCL12 did not transactivate HER1 in 5637 or HeLa cells.

### CXCL12 and its chemically synthesized mutant [N33A]CXCL12 regulate HER1 phosphorylation

Figure 3 shows a time-course of HER1 phosphorylation at Y1068 in HeLa (Figure 3A) and at Y1173 in 5637 (Figure 3B) cells after three different stimuli: 1) CXCL12 alone or 2) HB-EGF alone or 3) CXCL12 followed by stimulation with HB-EGF. Stimulation with CXCL12 alone induced no phosphorylation of HER1. With HB-EGF alone, HER1 phosphorylation reached a maximum at 1 minute, followed by partial dephosphorylation and a plateau at approximately 60% of maximal phosphorylation after 10 minutes. In contrast, cells prestimulated with CXCL12 showed a different pattern of HB-EGF-dependent HER1 phosphorylation. Treatment of cells with CXCL12 impacted HER1 phosphorylation in a typical way: the maximum degree of phosphorylation was reached later. Following stimulation with CXCL12, maximal phosphorylation was reached at 4 minutes, and the plateau after the initial spike was around 50% of the maximum after 10 minutes (Figure 3A, B). This delay in activation was not merely due to the more complex mechanistics of transactivation as opposed to direct stimulation with HB-EGF. Prestimulation with CXCL12 immediately followed by exogenous addition of HB-EGF to the cells led to the same phosphorylation pattern. Interestingly, the whole phenomenon was associated with a similar mitogenic effect of HB-EGF (Figure 3C), which suggested that CXCL12 sent inhibitory signals to HER1, which were transient in nature. In addition, we confirmed that the variant [N33A]CXCL12 had the same range of activity as the recombinant, non-variant form as far as CXCR4 stimulation, chemotactic activity and internalization were concerned [32]. However, prestimulation of the cells with 200 ng/mL of [N33A]CXCL12 for 1 minute inhibited HB-EGF-dependent phosphorylation of HER1. Whereas recombinant CXCL12 delayed the phosphorylation of HER1, [N33A]CXCL12 abolished it (Figure 3A, B).

### [N33A]CXCL12 acts as a G-protein-biased ligand

Figure 3D shows that downstream of CXCR4 the whole phosphorylation of ERK1/2 occurs in fact as a consequence of two different, sequentially ordered phosphorylation activities translating into two distinct ERK1/2 phosphorylation spikes: a precocious one that was G-protein-dependent *via* PKC and a late one that was β-arrestin-dependent. By using Ro-31, which inhibits PKC, recombinant CXCL12 induced only a late, β-arrestin-dependent ERK1/2 phosphorylation spike, but [N33A]CXCL12 induced no ERK1/2 phosphorylation at all. Therefore, [N33A]CXCL12 did not induce the β-arrestin-dependent phosphorylation of ERK1/2, while eliciting the G-protein-dependent spike. Panels E and F in Figure 3 show also that the inihibition of G-protein-dependent PKC with Ro-31 abolished the effect on HER1 phosphorylation at Y1068 and Y1173 induced by either recombinant or mutated CXCL12 prestimulation. Both the CXCL12-dependent delay and the [N33A]CXCL12-dependent full inhibition of HER1 phosphorylation seemed to be a G-protein-dependent phenomenon mediated *via* PKC.

### Blockade of β-arrestin 2 in 5637 and HeLa cells

If the variant form [N33A]CXCL12 induces full inhibition of HER1 for lack of β-arrestin activation, then recombinant CXCL12 should induce a full inhibition of HER1 in cells knockdown of β-arrestin. Therefore, to further test the transduction pathways downstream of CXCR4 we established a siRNA sequence targeted against the CXCR4-associated β-arrestin 2. This siRNA efficiently blocked β-arrestin in both 5637 and HeLa cells. Figure 4 shows that, when prestimulated with CXCL12, cells knockdown of β-arrestin 2 displayed no HB-EGF-dependent phosphorylation spikes and statistically significant less phosphorylation at HER1 Y1068 or Y1173 than their controls (p<0.05). Therefore, β-arrestin-silenced cells reacted to CXCL12 the way non-silenced cells reacted to [N33A]CXCL12, i.e. the prestimulation with CXCL12 resulted in a full inhibition of HB-EGF-dependent HER1 phosphorylation. These β-arrestin silencing data complement the ERK1/2 phosphorylation and Ro-31 blocking experiments and strengthen our experimetal evidence that 1) CXCL12 may send inhibitory signals to HER1 through a G-protein/PKC dependent pathway when β-arrestin is not activated and 2) the variant form [N33A]CXCL12 signals preferentially through G-proteins/PKC (as also suggested by using the Ro-31 inhibitor) and does not activate β-arrestin (as also suggested by the lack of the late, β-arrestin-dependent ERK1/2 phosphorylation spike). Finally, the same pattern of inhibition/restoration of HER1 phosphorylation was present in both 5637 and HeLa cells.

### CXCL12 transinhibits HER1 via PKC/calmodulin/calcineurin

There were a number of candidate pathways to mediate the inhibitory effect induced by both forms of CXCL12 downstream of G-proteins/PKC. However, it is known that calmodulin can be activated by any sustained Ca^2+^ flux, thus suggesting a mechanism by which known signaling participants can modulate HER1 signaling. After treatment with CXCL12 or [N33A]CXCL12, we observed that not only the PKC inhibitor Ro-31 (Figure 3E, F), but also the calmodulin inhibitor W-13 restored the HER1 phosphorylation pattern induced by HB-EGF alone either in 5637 or HeLa cells (Figure 5A, B). Downstream of calmodulin, the signal is divergent toward calcineurin and Ca^2+^-calmodulin-dependent protein kinase II (CaMKII). CsA, a specific inhibitor of calcineurin, prevented the CXCL12 effect and was capable of restoring the normal pattern of HER1 phosphorylation (Figure 5A, B). Calcineurin activity was therefore necessary for CXCL12 to modify the phosphorylation pattern of HER1. Figure 5C depicts the pattern of HER1 Y1068 and Y1173 phosphorylation at the plateau in 5637 or HeLa cells. Pretreatment with [N33A]CXCL12 was fully inhibitory at this time, but calmodulin and calcineurin inhibitors W-13 and CsA, respectively, restored HER1 phosphorylation. CaMKII inhibitor KN-62 did not restore the phosphorylation, and the whole process was independent from CaMKII, which was not activated by CXCL12 signaling (data not shown). In conclusion, specific modifications to the primary structure of CXCL12 may lead to a preferential or biased activation of G-protein-dependent pathways downstream of CXCR4, activation of calmodulin and calcineurin, and the complete inhibition of HER1 autophosphorylation.

**Figure 6:**
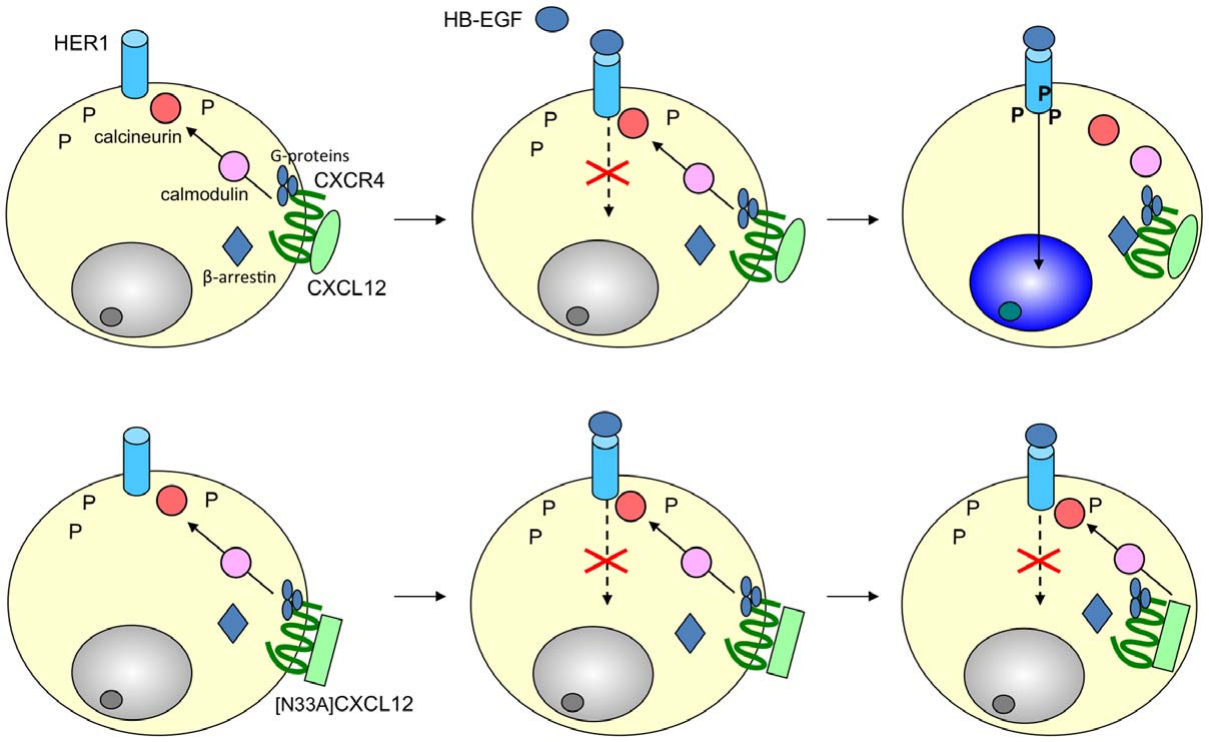
CXCL12 and [N33A]CXCL12 transinhibit HER1 in 5637 or HeLa cells. Both chemokines signal *via* G-proteins to calmodulin/calcineurin and modulate the ligand-dependent phosphorylation of HER1. CXCL12 induces a delay in the phosphorylation. [N33A]CXCL12, which is a G-protein-biased ligand, inhibits the HER1 phosphorylation.

## Discussion

The current study in 5637 and HeLa cells defines a novel mechanism of crosstalk between CXCR4 and HER1, whereby stimulation with recombinant CXCL12 modifies the pattern of HER1 phosphorylation *via* G-proteins/calmodulin/calcineurin. Beside, the study identifies [N33A]CXCL12 as a mutant of CXCL12 as a G-protein-biased CXCR4 ligand that induces a full inhibition of HER1 phosphorylation. Figure 6 gives a graphic synopsis of these findings. To our knowledge, there are no other studies showing that CXCL12 can transinhibit the phosphorylation of HER1 through intracytoplasmic pathways in CXCR4/HER1 double-positive cells.

Stimulation with CXCL12 led to 2 events: 1) activation of calmodulin/calcineurin and 2) transinhibition of tyrosine phosphorylation at the cytoplasmic tail of HER1. Prestimulation of HER1-positive target cells with CXCL12 modified HB-EGF-dependent HER1 stimulation by delaying peak phosphorylation at tyrosine 1068 and 1173. This effect was ultimately mediated by the Ca^2+^-calmodulin-activated protein phosphatase calcineurin, because PKC, calmodulin or calcineurin, but not CaMKII, inhibition could abolish the delay of the HER1 phosphorylation. All this did not affect CXCR4 internalization or CXCL12-dependent chemotaxis. Therefore the CXCL12-dependent inhibitory effect on HER1 was genuine, specific and associated with a G-protein/calmodulin/calcineurin-dependent transduction pathway. The variant [N33A]CXCL12 [28] induced CXCR4 internalization and chemotaxis in a manner similar to recombinant CXCL12 [32], but it abolished the HB-EGF-dependent Y1068 and Y1173 phosphorylation of HER1. It turned out that the variant CXCL12 acted as a G-protein-biased ligand for CXCR4, lacking the capability of activating β-arrestin-dependent ERK1/2 phosphorylation. Some [N33A]CXCL12-induced activities similar to recombinant CXCL12 may thus depend on p38 phosphorylation [33]. With both recombinant and variant forms, HER1 phosphorylation was restored by inhibiting PKC or calmodulin or calcineurin Though caution has been suggested when interpreting the effects obtained with calmodulin inhibitors in living cells, our results suggest in terms of exactly sequencial timing that it is the lack of β-arrestin ERK1/2 signaling that does maintain the inhibition of HER1 phosphorylation induced by [N33A]CXCL12. In other words, β-arrestin activation seems to revert HER1 transinhibition. We confirmed this in cells knockdown of β-arrestin in which recombinant CXCL12 led to full inhibition of HER1 as the mutant [N33A]CXCL12 did in non-silenced cells.

We demonstrate that in two different human neoplastic cell lines. But other studies provide circumstantial evidence that support and expand our findings, which are in line with experimental data regarding the effect of W-13 on HER1 activation [34], the global phosphorylation analysis downstream of β-arrestin [14], and β-arrestin-mediated HER1 transactivation [35]. Furthermore, in studying the CXCL12-induced phosphoproteome adjustments in Hodgkin's lymphoma-derived L540 cells, which do not express HER1 but other HER receptors, we found that HER2 phosphorylation is strongly downregulated 20 minutes after stimulation with CXCL12.

The CXCL12-mediated, calcineurin-dependent modulation of HER1 phosphorylation and the differential activity of recombinant and mutated molecules should be seen in the context of CXCR4 signaling specificity [36], [37]. In our experiments, activation of a receptor led to different biological effects depending on a single amino acid mutation in the ligand. The mutated amino acid was outside of the receptor binding domains, which shows that the ligand conformation provides a level of regulation for signaling specificity, in terms of biased agonism. In the present case, the biased agonist activated G-protein-dependent but not β-arrestin-dependent pathways and it was capable of exerting a full inhibitory effect on HER1 phosphorylation. This means that [N33A]CXCL12 influenced specifically both the magnitude and persistence of the cytokine signal as opposed to recombinant CXCL12, thus leading to differentially regulated cellular activities. In general, this conformation-related, magnitude- and persistence-dependent signaling should be evaluated in terms of cytokine networks that in turn either generate or diminish the specificity of a given signal [3], [38]. It may also have played a role in evolutionary terms [39], and we can envisage diseases arising from acquired or congenital variations of chemokine/receptor structure conditioning biased responses, as well as selective chemokine-derived drugs [17], [18]. Because there is at least one structural variant of CXCL12 that inhibits HER1 phosphorylation, it follows that we have devised a strategy in which CXCL12 variants may be sorted out in order to find out if and how they can regulate HER1 signaling. This in turn raises the question as to whether this may encompass a possible approach to control unwarranted proliferation *via* engineered CXCL12 with inhibitory capabilities.

The modulation of HER1 phosphorylation may play a role in cellular responses characteristic of invasive growth. This can be thought of as a behavioral program that regulates cell dissociation from its microenvironment, thus promoting cell migration and the colonization of new sites. This program is shared by normal cells involved in developmental, inflammatory or immune tasks and cancer cells in the metastatic process. Based on the findings in the present and other works [40], [41], [42], the crosstalk between CXCL12 and HERs adds a new layer to HERs regulation that may be involved in shifting cells alternatively toward differentiation (HER1 transinhibition) or self-renewal (HER1 transactivation). In addition to its direct action on neoplastic cells, CXCL12 has emerged as a monocyte-macrophage regulator in the context of the M2-polarized inflammatory responses [5]. M2-polarized tumor-associated macrophages may promote tumor cell proliferation under CXCL12 stimulation [5], [8]. Eventually this links inflammation to proliferation/differentiation and to oncogenes [8], [43], [44], [45], [46], [47]. Therefore, structural variants that transinhibit HER receptors should be tested to downregulate the mitogenic potential of CXCR4/HER double-positive neoplastic cells, to redirect the activity of CXCL12 on monocytes/macrophages and to potentiate M1 responses. This should be a more conservative and subtle approach as opposed to the use of CXCR4 inhibitors.

In conclusion, this work provides new evidence that the signaling mediated by CXCL12 *via* CXCR4 is capable of activating an inhibitory calmodulin/calcineurin module that modifies the magnitude and persistence of HER1 signaling. Taken together, these data contribute a refinement to our understanding of the signaling downstream of CXCR4 and HER1 [48]. This hints at a unifying concept for explaining the significance of the CXCL12/HER1 crosstalk in physiology and pathology dependent on the balance between transinhibition and transactivation, while suggesting at the same time a variety of potentially selective therapeutic tools in a number of reactive or neoplastic conditions.
